# Gender differences in psychosocial determinants of hand hygiene among physicians

**DOI:** 10.1017/ice.2023.199

**Published:** 2024-02

**Authors:** Se Yoon Park, Jaewoong Kim, Eunjung Lee, Sunghee Park, Jung-Wan Park, Shi Nae Yu, Tark Kim, Min Hyok Jeon, Eun Ju Choo, Tae Hyong Kim

**Affiliations:** 1 Department of Internal Medicine, Hanyang University College of Medicine, Seoul, Korea; 2 Department of Biomedical Systems Informatics, Yonsei University College of Medicine, Seoul, Korea; 3 Division of Infectious Diseases, Department of Internal Medicine, Soonchunhyang University Seoul Hospital, Soonchunhyang University College of Medicine, Seoul, Korea; 4 Division of Infectious Diseases, Department of Internal Medicine, Soonchunhyang University Bucheon Hospital, Bucheon, Korea; 5 Division of Infectious Diseases, Department of Internal Medicine, Soonchunhyang University Cheonan Hospital, Cheonan, Korea

## Abstract

**Objective:**

We investigated gender differences in psychosocial determinants that affect hand hygiene (HH) performance among physicians.

**Design:**

The survey included a structured questionnaire with 7 parts: self-assessment of HH execution rate; knowledge, attitude, and behavior regarding HH; internal and emotional motivation for better HH; barriers to HH; need for external reminders; preference for alcohol gel; and embarrassment due to supervision.

**Setting:**

The study was conducted across 4 academic referral hospitals in Korea.

**Participants:**

Physicians who worked at these hospitals were surveyed.

**Methods:**

The survey questionnaire was sent to 994 physicians of the hospitals in July 2018 via email or paper. Differences in psychosocial determinants of HH among physicians were analyzed by gender using an independent *t* test or the Fisher exact test.

**Results:**

Of the 994 physicians, 201 (20.2%) responded to the survey. Among them, 129 (63.5%) were men. Male physicians identified 4 barriers as significant: time wasted on HH (*P* = .034); HH is not a habit (*P* = .004); often forgetting about HH situations (*P* = .002); and no disadvantage when I do not perform HH (*P* = .005). Female physicians identified pain and dryness of the hands as a significant obstacle (*P* = .010), and they had a higher tendency to feel uncomfortable when a fellow employee performed inadequate HH (*P* = .098). Among the respondents, 26.6% identified diversifying the types of hand sanitizers as their first choice for overcoming barriers to improving HH, followed by providing reminders (15.6%) and soap and paper towels in each hospital room (13.0%).

**Conclusion:**

A significant difference in the barriers to HH existed between male and female physicians. Promoting HH activities could help increase HH compliance.

Hand hygiene (HH) is an essential aspect of infection control in healthcare settings as it prevents the spread of hospital-acquired infections.^1,2^ Healthcare-associated infections can lead to significant morbidity, mortality, and healthcare costs.^3^ Despite its importance, compliance with HH among healthcare workers is often suboptimal, and various factors contribute to this issue.^4,5^ These factors vary at individual, team, and institutional levels; understanding the barriers to HH performance at each level and developing effective strategies to improve HH compliance are critical for reducing healthcare-associated infections.^6^

The importance of HH has been highlighted in Korea following the 2009 influenza pandemic and the 2015 Middle East respiratory syndrome coronavirus outbreak. A nationwide survey conducted every 2 years since 2013 has shown that institutional awareness of HH has improved.^7^ In addition, a feasibility study conducted in 2016 showed that Korean hospitals spent ∼$3,000 and ∼$4,000 per 100 beds annually for hand sanitizers and soap, respectively. However, only 46.5% of physicians practiced HH, compared to 55.5% of nurses and 49.4% of medical technicians.^8^ Moreover, our previous study showed that male physicians had low compliance rates in HH performance than women.^9^

Several studies have shown gender differences in compliance with HH among healthcare workers; men generally have lower compliance rates than women.^9–11^ In particular, gender differences in HH have been frequently reported outside healthcare settings; however, the underlying reasons for these differences remain unclear.^12,13^ Understanding the factors that contribute to gender differences in HH is crucial for developing effective interventions that improve compliance and reduce the risk of healthcare-associated infections. However, few studies have explored the factors responsible for the gender differences in the practice of HH.

Therefore, we investigate the knowledge, attitudes, and motivation for HH among physicians and to identify barriers to HH practice and interventions to overcome them. In this study, we analyzed gender differences to understand the causes of gender differences in HH practice.

## Methods

### Study design

We conducted a survey on HH among physicians at 4 referral Soonchunhyang University–affiliated hospitals located in Korea. One hospital is located in the metropolitan area of Seoul (hospital A), and one hospital is located in a city adjacent to Seoul, Bucheon (hospital B). The other 2 hospitals are in rural areas, namely Cheonan (hospital C) and Gumi (hospital D). The survey targets all physicians working in these hospitals. At the time of the survey, 994 physicians were identified as potential participants (Supplementary Table 1 online). The survey was sent via e-mail or paper to the infection control team of each hospital. The survey was conducted over a period of 14 days (July 9–22, 2018). To encourage participation, reminders were sent by the infection control team on days 4, 7, and 10 of the survey. The survey included a structured questionnaire with 7 parts: (1) self-reported HH and optimal compliance rate, (2) knowledge, attitude, and behavior regarding HH (11 questions), (3) internal and emotional motivation for better HH (11 questions), (4) barriers for HH (14 questions), (5) the need for external supervision (4 questions), (6) preference for alcohol gel (3 questions), and (7) embarrassment due to supervision (2 questions). HH compliance was determined by dividing the number of observed HH actions by the total number of opportunities based on participants’ self-assessed adherence. Optimal HH compliance rates were calculated based on self-assessed adherence to the 6-step technique and the appropriate time recommended by the World Health Organization (WHO).^1^

### Survey items

The survey instrument was adapted from a previous study conducted by Ibrahim et al,^14^ which was based on WHO knowledge and perception surveys on HH and focus group interviews. Additionally, strategies for overcoming barriers to HH have also been developed.^14^

Compliance rates with HH and optimal HH were developed as self-reported items from the WHO recommendations.^15^ The Five Moments are delineated as follows: (1) before touching a patient, (2) before clean or aseptic procedure, (3) after body fluid exposure risk, (4) after touching a patient, and (5) after touching a patient’s surroundings. The 6-step technique consists of (1) rubbing hands palm to palm, (2) interlocking fingers and rub the back of fingers of both hands, (3) rubbing the back of both hands, (4) rubbing thumb in a rotating manner followed by the area between index finger and thumb for both hands, (5) interlacing fingers and rub hands together, and (6) rubbing fingertips on palm for both hands.

The items for importance and achievement related to HH were adapted from a previous study.^16^ We selected 10 HH promotion activities currently implemented or potentially implemented in the future: (1) hand sanitizer placed where necessary, (2) regular HH education, (3) practical training according to the situation, (4) frequent monitoring, (5) department-wide feedback, (6) personal feedback, (7) HH information posters, (8) audiovisual alarm or guidance, (9) management’s interest and encouragement, and (10) rewarding and publicizing excellent HH employees and departments. Each item consisted of a Likert 5-point scale, and data were collected in the form of evaluating the importance and achievement of the same item. Importance referred to the importance of the HH promotion activity in the respondent’s opinion, whereas achievement referred to the actual implementation of the activity in the study hospitals. The other items in the HH survey consisted of 14 items rated on a 7-point Likert scale. The higher the score, the higher the degree of agreement. The method for improving HH was configured such that the respondents selected 3 methods to improve HH and indicated the order of their preference among the total 12 selected items.

### Statistical analysis

Statistical analyses were performed using the R version 4.2.2 software (https://www.r-project.org). Descriptive analysis was performed by calculating frequencies and percentages. The comparisons among study hospitals, positions, age groups, and measures for overcoming barriers to performing HH were analyzed using the χ^2^ or Fisher exact test. The differences in self-reported HH, internal and external motivations, and barriers were assessed by gender using an independent *t* test. *P* values <.05 were considered statistically significant.

### Ethics approval

This study was approved by the Institutional Review Board of Soonchunhyang University Seoul Hospital (no. 2019-01-008).

## Results

**Figure 1.**
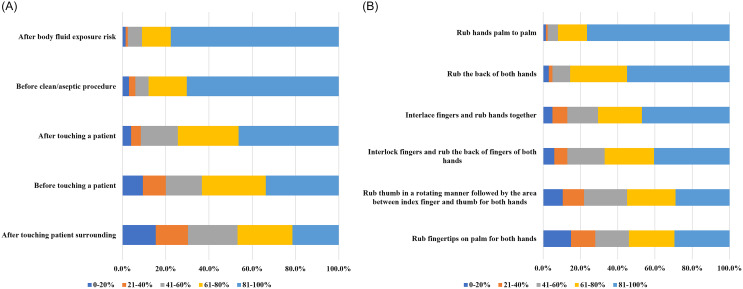
Self-reported hand hygiene compliance rates according to (A) the Five Moments and (B) the six-step technique.

Of the 994 physicians in the 4 hospitals, 201 (20.2%) completed the survey. Among them, 129 (63.5%) were men. The respondents’ demographic characteristics are presented in Table 1. The self-reported HH and optimal HH compliance rates were 75.5% and 57.6%, respectively. Self-reported compliance to HH and optimal HH was lower in men than in women, but this difference was not statistically significant (*P* = .202 for HH and .638 for optimal HH). A similar pattern was observed in hospitals A, B, and C. In contrast, both self-reported HH and optimal HH compliance were higher in men than in women at hospital D (Supplementary Table 2 online). Self-reported compliance to HH at the Five Moments was highest in “after body fluid exposure risk,” followed by “before clean/aseptic procedure,” “after touching a patient,” “before touching a patient,” and “after touching patient surrounding.” With respect to the 6 steps, self-reported HH compliance was highest in “rub hands palm to palm” and lowest in “rub thumb or fingertips” (Fig. 1).

**Table 1.** Characteristics of Study Participants Stratified by Gender

Categories	Total (N = 201), No. (%)	Male (N = 129), No. (%)	Female (N = 72), No. (%)	*P* Value
**Hospital**				
A	56 (27.9)	38 (67.9)	18 (32.1)	.023
B	67 (33.0)	34 (50.7)	33 (49.3)	
C	51 (25.4)	35 (68.6)	16 (31.4)	
D	27 (13.3)	22 (81.5)	5 (18.5)	
**Occupation**				
Intern	28 (13.9)	19 (67.9)	9 (32.1)	.040
Resident	59 (29.4)	45 (76.3)	14 (23.7)	
Professor	114 (56.7)	65 (57.0)	49 (43.0)	
**Age group**				
20–29 y	55 (27.8)	40 (72.7)	15 (27.3)	.032
30–39 y	76 (38.4)	42 (55.3)	34 (44.7)	
40–49 y	50 (25.3)	31 (62.0)	19 (38.0)	
50–59 y	17 (8.6)	15 (88.2)	2 (11.8)	
Allergy to alcohol hand sanitizer	15 (7.9)	8 (53.3)	7 (46.7)	.312

Note. Percentages for the total column report column percentages and for the male and female columns report row percentages.

### Importance and achievement of HH promotion activities

**Figure 2.**
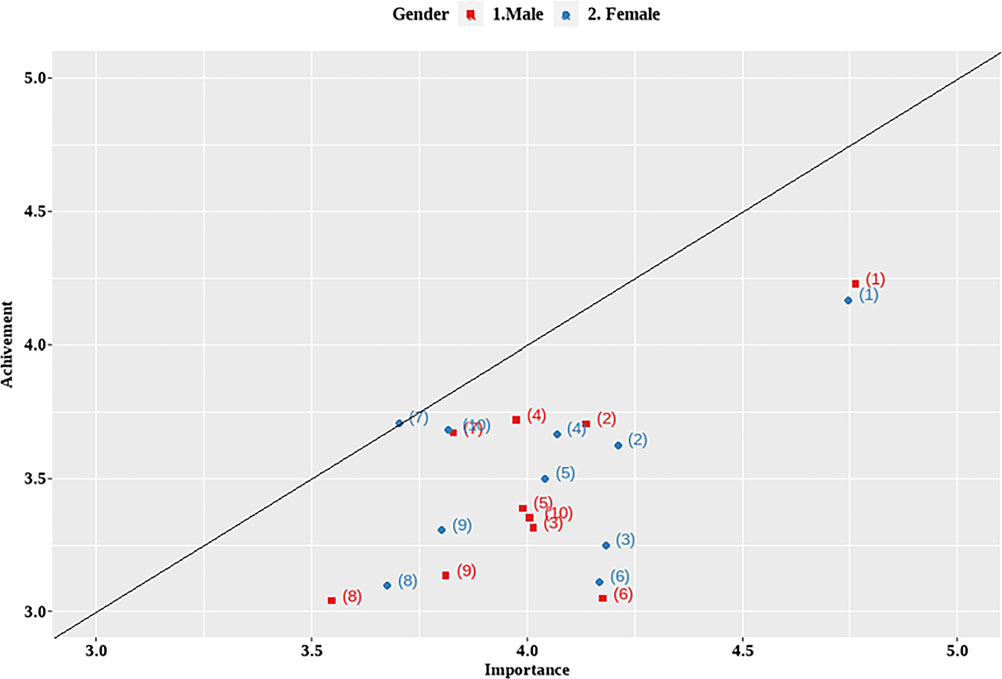
Relationship between importance and achievement. (1) Hand sanitizer placed where necessary, (2) regular hand hygiene education, (3) practical training according to the situation, (4) frequent monitoring, (5) department-wide feedback, (6) personal feedback, (7) hand hygiene information poster, (8) audiovisual alarming/guidance, (9) management’s interest and encouragement, and (10) reward and publicize excellent hand hygiene employees and departments.

Figure 2 and Supplementary Table 3 (online) show the relationship between importance and achievement. The importance scores were higher than the achievement scores for both men and women. The most important factor promoting HH compliance was “hand sanitizer placed where necessary,” followed by “personal feedback” and “regular HH education.” The rank of importance in the male group was the same as the overall rank. In the female group, the rank of importance was “hand sanitizer placed where necessary,” “regular HH education,” and “practical training according to the situation.” The largest gap was seen between importance and achievement in “personal feedback.”

Most physicians received HH education once a year, with education provided by the infection control team being the most common. Female physicians received education more frequently than male physicians, although the difference was not statistically significant. Among the respondents, 32% indicated that they had not received any online education, and 28.4% reported not having received education within their department (Supplementary Table 4 online).

### Knowledge, attitude, and behavior regarding HH

There was no significant difference between male and female physicians in terms of knowledge, attitude, and behavior regarding HH, except for the statement “I perform HH before patient contact” (Supplementary Table 5 online).

### Barriers to HH

Among 14 barriers to HH, the item with the highest mean score was “HH is difficult in an emergency” with a score of 4.74, followed by “HH causes pain and dryness of hands (skin problems)” with a score of 3.87 and “I find it hard to tell a colleague to do HH” with a score of 3.66. Also, 5 items showed statistically significant differences between men and women: “HH causes pain and dryness of hands (skin problems),” “Time that could be spent on something more important is wasted on HH,” “HH has not become a habit,” “I often forget about HH,” and “I do not perform HH because there is no disadvantage when I do not perform it.” Among these 5 items, only scores for “HH causes pain and dryness of hands (skin problems)” were higher among women compared with men (Table 2).

**Table 2.** Comparison of Barriers to Practicing Hand Hygiene Among Male and Female Physicians

Questions	Total, Mean ±SD	Male, Mean ±SD	Female, Mean ±SD	*P* Value
Hand hygiene is difficult in an emergency.	4.74±1.62	4.87±1.66	4.51±1.53	.129
Hand hygiene causes pain and dryness of hands (skin problems).	3.87±1.85	3.62±1.84	4.32±1.80	.010
I find it hard to tell a colleague to perform hand hygiene.	3.66±1.60	3.73±1.59	3.54±1.61	.429
I often forget about hand hygiene situations.	3.34±1.64	3.60±1.65	2.89±1.52	.002
It is hard to perform hand hygiene when a superior does not perform hand hygiene.	3.30±1.76	3.41±1.83	3.11±1.63	.234
It is hard to perform hand hygiene because soap and hand towels are not prepared in every hospital room.	3.09±1.62	3.12±1.65	3.06±1.57	.797
Hand hygiene is not a habit.	3.01±1.64	3.26±1.65	2.58±1.54	.004
I do not want to perform hand hygiene when I am being observed.	2.97±1.77	3.08±1.84	2.78±1.62	.233
Time that could be spent on something more important is wasted on hand hygiene.	2.96±1.52	3.12±1.60	2.67±1.32	.034
I do not perform hand hygiene because there is no disadvantage when I do not perform it.	2.83±1.68	3.06±1.76	2.42±1.43	.005
Hand hygiene is not needed if you wear gloves.	2.75±1.60	2.79±1.62	2.67±1.56	.595
There is no ethical problem with not performing hand hygiene.	2.32±1.55	2.42±1.57	2.15±1.50	.238
I do not know exactly when to perform hand hygiene.	2.32±1.45	2.43±1.54	2.12±1.26	.125
Hand hygiene has no clear relation to patient safety.	2.09±1.47	2.22±1.57	1.86±1.24	.072

Note. SD, standard deviation.

### Internal and emotional motivation

There were no significant differences between male and female physicians in the 8 questions on internal motivation. Male physicians were more motivated to get a promotion than female physicians, but the difference was not statistically significant. Female physicians had a higher tendency to feel uncomfortable when their fellow employees performed inadequate HH (*P* = .098) (Table 3).

**Table 3.** Comparison of Internal and Emotional Motivation for Hand Hygiene Among Male and Female Physicians

Questions	Total, Mean ±SD	Male, Mean ±SD	Female, Mean ±SD	*P* Value
**Internal motivation**				
I can do better hand hygiene if the sink is near.	5.25±1.32	5.29±1.31	5.19±1.33	.860
Soap or hand towels are provided in each hospital room for good hand hygiene.	5.05±1.56	5.14±1.52	4.93±1.6	.370
I want to receive feedback on hand hygiene and improve my performance.	5.01±1.24	5.09±1.24	4.9±1.26	.486
If I could get a promotion for hand hygiene, I would do better for hand hygiene.	4.92±1.65	5.08±1.63	4.69±1.61	.163
Our hospital staff regularly receive feedback on hand hygiene practices.	4.9±1.39	4.92±1.41	4.9±1.31	.870
Hand hygiene is most important in my work.	4.63±1.57	4.64±1.66	4.67±1.37	.580
Hand hygiene posters and screensavers help with hand hygiene.	4.59±1.44	4.69±1.46	4.43±1.4	.279
I do hand hygiene to become a role model to my colleagues.	4.32±1.68	4.32±1.69	4.36±1.66	.646
**Emotional motivation**				
I feel guilty when I do not perform hand hygiene.	4.36±1.50	4.23±1.52	4.58±1.45	.113
I feel embarrassed when a supervisor tells me to perform hand hygiene.	3.89±1.64	3.97±1.65	3.74±1.60	.334
I feel uncomfortable when a fellow employee performs inadequate hand hygiene.	3.86±1.51	3.73±1.53	4.10±1.47	.098

Note. SD, standard deviation.

### Improvements to overcome barriers to HH

The overall preference for the improvement strategies was ranked in the order of “diversify types of hand sanitizers,” followed by “remind timing of HH through a reminder” and “install soap and paper towels in each hospital room.” The percentage distribution on the choice of improvement strategies in women was of the same order as the overall distribution, and in men, the first 2 choices were the same, whereas the third was “change perception through various HH campaigns” (Table 4).

**Table 4.** Measures for Overcoming Barriers to Performing Hand Hygiene

Method	Total, No. (%)	Male, No. (%)	Female, No. (%)	*P* Value
Diversify types of hand sanitizers.	51 (26.6)	29 (23.4)	22 (32.4)	.281
Remind timing of hand hygiene through a reminder.	30 (15.6)	19 (15.3)	11 (16.2)	
Induce an atmosphere of requesting hand hygiene from staff through patient and caregiver education	25 (13.0)	15 (12.1)	10 (14.7)	
Change perception through various hand hygiene campaigns.	24 (12.5)	18 (14.5)	6 (8.8)	
Hand hygiene results are reflected in the personal review.	18 (9.4)	13 (10.5)	5 (7.4)	
Provide on the spot feedback of the observation.	14 (7.3)	11 (8.9)	3 (4.4)	
Monitoring is carried out on a regular basis.	11 (5.7)	6 (4.8)	5 (7.4)	
Install soap and paper towels in each hospital room.	7 (3.6)	7 (5.6)	0 (0.0)	
Hand hygiene real-name system to manage personal performance rate.	6 (3.1)	4 (3.2)	2 (2.9)	
Conduct a peer-to-peer assessment of performance rates.	4 (2.1)	2 (1.6)	2 (2.9)	
Strengthen hand hygiene theory education.	1 (0.5)	0 (0.0)	1 (1.5)	
Practical training for strengthening hand hygiene compliance.	1 (0.5)	0 (0.0)	1 (1.5)	

The need for external reminders (ie, “I sometimes forget about HH”) was higher in men than in women, but the difference was statistically nonsignificant: the mean was 3.91 in men versus 3.53 in women (*P* = .096). The highest score was observed for a colleague’s reminder, followed by exemplary leadership, and was observed in HH monitoring (Supplementary Table 6 online). Male physicians showed higher scores regarding “preference for alcohol gel hand sanitizer” than those of women, but the difference was statistically nonsignificant (Supplementary Table 7 online).

## Discussion

We investigated the differences in HH between male and female physicians, and we explored the reasons for these differences. Male physicians tended to have a lower rate of self-reported HH compliance than female physicians and faced different barriers to performing HH. Male physicians reported barriers related to time, habit, forgetfulness, and lack of disadvantage when not performing HH. In contrast, female physicians reported increased skin problems as a significant barrier to HH compliance. These findings suggest that interventions to improve HH should be tailored to the specific barriers faced by male and female physicians.

Moreover, this study highlights the importance of addressing the emotional and internal motivations behind HH compliance. Male physicians were more motivated by the prospect of obtaining a promotion, and female physicians tended to feel uncomfortable when a fellow employee did not adequately perform HH. However, these findings were not statistically significant and must be confirmed in another study with a larger sample size.

In this study, we identified difficulty in performing HH in an emergency situation as the top barrier for both male and female physicians. There is a strong association between hospital-acquired infections and procedures such as central venous catheterization, intubation, and urinary catheterization, which are commonly performed in emergency situations.^17^ Moreover, admission to the emergency room increases the risk of hospital-acquired infections.^18^ Therefore, it is crucial to maintain proper HH, even in emergency situations, to prevent the transmission of other diseases or infections in patients.

Among male physicians, “I find it hard to tell a colleague to perform HH” was identified as the second highest barrier. It is generally difficult to tell a colleague to perform HH. However, our prior research found that among physicians, leadership has an important influence on followers performing HH.^9^ In another study, the HH performance of a senior or the first person entering the room during rounds influenced the HH of the rest.^19^ Therefore, strategies to improve HH performance should consider these aspects.

In addition, HH causes pain and dryness of the hands in women, which is a significant barrier. In a survey, ∼25% of nurses reported symptoms or signs of hand dermatitis, and as many as 85% said they had experienced skin problems.^20^ Healthcare workers should be provided with information to reduce the risk of contact dermatitis and skin damage. They should replace potentially irritating products with formulations that cause less skin damage. Furthermore, staff should be educated about the risks of irritant contact dermatitis. Lotions or creams should also be used to minimize dryness and irritation of the hands. Washing hands with soap and water immediately after using a hand sanitizer can cause dermatitis; therefore, employees should be reminded that regular hand washing is neither necessary nor recommended after using an alcoholic hand sanitizer.^21^

Despite the lack of statistical significance, male physicians received HH education less frequently than their female counterparts, indicating a potential disparity in participation rates. To address this issue, it is advisable to provide increased educational opportunities, including online lectures, workshops, and awareness campaigns, to enhance male physicians’ familiarity with HH practices.

The achievement of HH promotion activities fell short of their importance among both male and female physicians, highlighting the need for greater institutional intervention. Improving the accessibility of hand sanitizers received the highest scores in terms of importance and achievement. Although HH gel was available at every bedside during the survey period, it was evident that more accessibility was necessary. Notably, however, this survey was conducted before the COVID-19 pandemic, and the results may have been different if the survey had been conducted after the pandemic. The most significant disparity between importance and achievement was observed in the individual feedback. To address this gap, providing individual feedback to physicians is crucial.

This study had several limitations. The sample size was small, and we focused on physicians alone, which may not reflect the HH of all healthcare workers. Additionally, possible biases due to the low response rate of the survey should be considered in interpreting the results. Secondly, the barriers to HH compliance and measures to overcome them can be different after the COVID-19 pandemic. Lastly, the study relied on self-reported HH compliance rates, which may have been subject to social desirability bias.

In conclusion, our findings provide contribute to the design of effective HH compliance campaigns. Given that male and female healthcare workers face different barriers to HH compliance, campaigns should be tailored to address the specific challenges faced by each group. The findings from this study can be applied to develop studies to understand the practices of all healthcare workers.

## Supplementary material

For supplementary material accompanying this paper visit https://doi.org/10.1017/ice.2023.199. click here to view supplementary material
