# Supplementary material for: Gender differences in psychosocial determinants of hand hygiene among physicians
Source: Infect Control Hosp Epidemiol. 2023 Oct 4;45(2):215–20. doi: 10.1017/ice.2023.199 (PMC10877538; doi:10.1017/ice.2023.199)
Supplement: Supplementary file 1 [file S0899823X2300199Xsup001.docx]

**Supplemental Table 1.** Distribution of potential participants and response rate by position among physicians

|  | Professor | Resident | Intern | Total |
| --- | --- | --- | --- | --- |
| Hospital A | 166 (55.5) | 122 (37.4) | 21 (7.2) | 299 |
| Hospital B | 195 (60.7) | 98 (30.5) | 28 (8.8) | 321 |
| Hospital C | 174 (62.2) | 88 (31.7) | 17 (6.2) | 279 |
| Hospital D | 65 (69.1) | 18 (19.1) | 11 (11.8) | 94 |
| Total | 600 (60.3) | 316 (31.8) | 78 (7.9) | 994 |
| Responders | 114 (56.7) | 59 (29.4) | 28 (13.9) | 201 |
| Non-responders | 486 (61.3) | 257 (32.4) | 50 (6.3) | 793 |
| Response rate, % | 19 (114/600) | 18.7 (59/316) | 35.9 (28/78) | 20.2 (201/994) |

Data are expressed as numbers (%) unless otherwise indicated.

**Supplemental Table 2.** Self-reported hand hygiene compliance rates according to gender and study hospital

| Hospital | Variables | Total | Male | Female | *P* value |
| --- | --- | --- | --- | --- | --- |
| Total | Hand hygiene compliance | 75.50±19.80 | 74.22±19.72 | 77.94±19.92 | 0.202 |
|  | Optimal hand hygiene compliance | 57.64±19.78 | 56.79±19.72 | 58.68±19.92 | 0.638 |
| Hospital A | Hand hygiene compliance | 74.89±17.79 | 73.03±13.93 | 78.00±24.27 | 0.334 |
|  | Optimal hand hygiene compliance | 52.28±24.82 | 50.13±25.38 | 56.39±24.42 | 0.387 |
| Hospital B | Hand hygiene compliance | 75.25±18.55 | 71.76±20.07 | 78.85±16.36 | 0.119 |
|  | Optimal hand hygiene compliance | 59.04±28.25 | 54.15±31.87 | 64.09±23.40 | 0.150 |
| Hospital C | Hand hygiene compliance | 71.98±22.84 | 71.29±22.57 | 74.25±24.56 | 0.674 |
|  | Optimal hand hygiene compliance | 51.44±29.64 | 51.43±29.27 | 49.06±30.67 | 0.793 |
| Hospital D | Hand hygiene compliance | 84.52±18.75 | 84.73±20.70 | 83.60±5.90 | 0.906 |
|  | Optimal hand hygiene compliance | 77.41±22.84 | 80.91±20.27 | 62.00±29.50 | 0.095 |

Data are expressed as mean ± standard deviation.

**Supplemental Table** **3.** Relationship between importance and achievement

| Variables | Category | Total | Male | Female | *P* value |
| --- | --- | --- | --- | --- | --- |
| Hand sanitizer placed where necessary | Importance | 4.76±0.47 | 4.76±0.46 | 4.75±0.47 | 0.798 |
|  | Achievement | 4.20±0.75 | 4.23±0.71 | 4.17±0.82 | 0.590 |
| Regular hand hygiene education | Importance | 4.15±0.78 | 4.14±0.79 | 4.21±0.74 | 0.526 |
|  | Achievement | 3.68±0.85 | 3.70±0.87 | 3.63±0.83 | 0.537 |
| Practical training according to the situation | Importance | 4.07±0.84 | 4.02±0.87 | 4.18±0.76 | 0.181 |
|  | Achievement | 3.29±0.95 | 3.31±0.98 | 3.25±0.92 | 0.658 |
| Frequent monitoring | Importance | 4.01±0.85 | 3.98±0.89 | 4.07±0.78 | 0.456 |
|  | Achievement | 3.71±0.88 | 3.72±0.84 | 3.67±0.93 | 0.687 |
| Department-wide feedback | Importance | 4.01±0.83 | 3.99±0.88 | 4.04±0.75 | 0.672 |
|  | Achievement | 3.43±0.97 | 3.39±0.99 | 3.50±0.93 | 0.427 |
| Personal feedback | Importance | 4.17±0.75 | 4.18±0.79 | 4.17±0.70 | 0.940 |
|  | Achievement | 3.08±1.00 | 3.05±1.01 | 3.11±0.99 | 0.667 |
| Hand hygiene information poster | Importance | 3.79±0.98 | 3.83±1.03 | 3.70±0.90 | 0.388 |
|  | Achievement | 3.68±0.92 | 3.67±0.95 | 3.71±0.88 | 0.775 |
| Audiovisual alarming/guidance | Importance | 3.59±1.02 | 3.55±1.06 | 3.68±0.95 | 0.403 |
|  | Achievement | 3.06±1.06 | 3.04±1.08 | 3.10±1.04 | 0.710 |
| Management’s interest and encouragement | Importance | 3.81±1.07 | 3.81±1.13 | 3.80±0.97 | 0.949 |
|  | Achievement | 3.20±1.06 | 3.13±1.13 | 3.31±0.93 | 0.269 |
| Reward and publicize excellent hand hygiene employees/departments | Importance | 3.92±0.91 | 4.01±0.91 | 3.82±0.88 | 0.154 |
|  | Achievement | 3.47±0.96 | 3.35±0.98 | 3.68±0.92 | 0.020 |

Data are expressed as mean ± standard deviation.

**Supplemental Table 4.** Frequency of hand-hygiene education

| Education provider | Sex | No | Once or twice a year | Ones or twice a quarter | Ones or twice a month | Ones or twice a week | Total |
| --- | --- | --- | --- | --- | --- | --- | --- |
| Education within each department^a^ | Male | 38 (29.5) | 76 (58.9) | 11 (8.5) | 2 (1.6) | 2 (1.6) | 129 |
|  | Female | 19 (26.4) | 39 (54.2) | 12 (16.7) | 2 (2.8) | 0 | 72 |
|  | Total | 57 (28.4) | 115 (57.2) | 23 (11.4) | 4 (2.0) | 2 (1.0) | 201 |
| Education by infection control team^b^ | Male | 22 (17.2) | 85 (66.4) | 17 (13.3) | 3 (2.3) | 1 (0.8) | 128 |
|  | Female | 9 (12.7) | 44 (62.0) | 14 (19.7) | 3 (4.2) | 1 (1.4) | 71 |
|  | Total | 31 (15.6) | 129 (64.8) | 31 (15.6) | 6 (3.0) | 2 (1.0) | 199 |
| On-line education for entire employees^c^ | Male | 40 (31.3) | 72 (56.3) | 12 (9.4) | 2 (1.6) | 2 (1.6) | 128 |
|  | Female | 24 (33.3) | 37 (51.4) | 8 (11.1) | 1 (1.4) | 2 (2.8) | 72 |
|  | Total | 64 (32.0) | 109 (54.5) | 20 (10.0) | 3 (1.5) | 4 (2.0) | 200 |

^a^ Fisher’s exact test for sex differences, *P* = 0.354

^b^ Fisher’s exact test for sex differences, *P* = 0.553

^c^ Fisher’s exact test for sex differences, *P* = 0.920

**Supplemental** **Table 5.** Differences in knowledge, attitudes, and behaviors regarding hand hygiene according to se

| Questions | Total | Male | Female | *P* value |
| --- | --- | --- | --- | --- |
| I am at risk for infection if I do not perform hand hygiene. | 6.38±0.94 | 6.34±1.00 | 6.44±0.82 | 0.433 |
| Hand hygiene is a part of the treatment process. | 6.36±0.89 | 6.34±0.93 | 6.38±0.83 | 0.812 |
| Jewels and artificial nails aggravate bacterial colonization. | 6.34±0.85 | 6.31±0.84 | 6.39±0.87 | 0.542 |
| I know when to perform hand hygiene. | 6.34±0.71 | 6.33±0.67 | 6.35±0.79 | 0.895 |
| I know how to perform hand hygiene correctly. | 6.33±0.75 | 6.35±0.70 | 6.29±0.83 | 0.605 |
| I believe hand hygiene prevents transmission of infections. | 6.32±0.90 | 6.26±0.97 | 6.44±0.73 | 0.179 |
| Hand hygiene is important in preserving my professional expertise. | 6.30±0.91 | 6.35±0.84 | 6.21±1.02 | 0.293 |
| Patients expect me to perform appropriate hand hygiene. | 6.14±0.96 | 6.08±1.01 | 6.25±0.87 | 0.226 |
| Performing hand hygiene is convenient because hand sanitizer is nearby. | 6.07±1.08 | 6.02±1.04 | 6.15±1.15 | 0.416 |
| I perform hand hygiene before patient contact. | 5.82±1.13 | 5.67±1.16 | 6.07±1.03 | 0.017 |
| Hand hygiene performance of my colleagues affects my hand hygiene performance rate. | 5.71±1.12 | 5.68±1.19 | 5.78±1.22 | 0.571 |

Data are expressed as mean ± standard deviation.

**Supplemental Table 6.** Need for external reminders

| Variables | Total | Male | Female | *P* value |
| --- | --- | --- | --- | --- |
| If your colleagues tell you to do hand hygiene, your compliance will go up. | 4.87±1.38 | 4.92±1.45 | 4.79±1.26 | 0.524 |
| The compliance rate will go up if the hospital's administrators, professors, team leaders do a good job of hand hygiene. | 4.86±1.46 | 4.80±1.51 | 4.97±1.37 | 0.44 |
| If someone is monitoring hand hygiene, compliance will go up. | 4.80±1.40 | 4.73±1.49 | 4.93±1.21 | 0.323 |
| I sometimes forget about hand hygiene. | 3.77±1.54 | 3.91±1.55 | 3.53±1.50 | 0.096 |

Data are expressed as mean ± standard deviation.

**Supplemental Table 7.** Preference for alcohol gel hand sanitizer

| Variables | Total | Male | Female | *P* value |
| --- | --- | --- | --- | --- |
| Alcohol gel hand sanitizer cleans my hands. | 5.31±1.19 | 5.34±1.24 | 5.25±1.10 | 0.604 |
| I prefer alcohol gel hand sanitizer to water and disinfectant soap. | 4.90±1.54 | 5.01±1.52 | 4.69±1.57 | 0.168 |
| I like the alcohol gel hand sanitizer in our hospital. | 4.83±1.44 | 4.96±1.42 | 4.58±1.46 | 0.075 |

Data are expressed as mean ± standard deviation.
